# User experience of controlling the DEKA Arm with EMG pattern recognition

**DOI:** 10.1371/journal.pone.0203987

**Published:** 2018-09-21

**Authors:** Linda J. Resnik, Frantzy Acluche, Shana Lieberman Klinger

**Affiliations:** 1 Research Department, Providence VA Medical Center, Providence, Rhode Island, United States of America; 2 Health Services, Policy and Practice, Brown University, Providence, Rhode Island, United States of America; University of Chicago, UNITED STATES

## Abstract

**Introduction:**

A commercially available EMG Pattern Recognition (EMG-PR) control system was adapted to interface with the multi-degree of freedom (DOF) DEKA Arm.

**Purpose:**

To describe users’ experience of controlling the DEKA Arm using EMG-PR.

**Methods:**

Sample: Twelve persons with upper limb amputation participated, 10 with transradial (TR), 2 with transhumeral (TH) level amputation. Ten were male, and 11 were users of a prosthesis at baselines. Design: This was a two-part study consisting of in-laboratory prosthetic training (Part A) and up to 12 weeks of home use of the prosthesis (Part B). Data collection: Qualitative data were collected through open-ended survey questions and semi-structured interviews. Data Analysis: The study used a qualitative case series design with a constant comparative approach to identify common categories of experience. Coding categories were iteratively refined until saturation of categories was achieved. The data were organized in a primary category, major categories of experience, factors impacting experience, and broader contextual factors.

**Results:**

Users had mixed perspectives on the desirability of the EMG-PR control system in combination with the DEKA Arm. Major aspects of user experience related to the system complexity, process of calibrating, and functional benefits. Factors influencing user experience included training and acclimation, fatigue, prosthesis design, technical issues and control changes. Broader contextual factors, both personal and environmental, also impacted users’ experience.

**Discussion/Conclusion:**

This study provided an in-depth description of user experience operating the DEKA Arm using EMG-PR control. The majority of participants expressed a preference for the controls of their personal prosthesis and controls rather than the iteration of EMG-PR controlled DEKA Arm used in this study. Most were positive about the future potential of EMG-PR as a control method. An understanding of patient experience will assist clinicians and patients choosing prosthetic options.

## Introduction

Upper limb prostheses have advanced in the past decade, with the commercial release of devices that have multiple powered degrees of freedom (DOF). While these devices enable more movements that more closely resemble the movements of the anatomical limb, they are challenging to control. Historically, powered upper limb prostheses have been controlled using dual myoelectric (EMG) inputs. Each input controls a single direction of movement, and switching between joints is accomplished by using an alternate signal. Another option for prosthetic control is foot control using inertial measurement units (IMU) [1]. The use of IMU controls has been spearheaded in the DEKA Arm, an FDA approved upper limb prosthesis that has recently become commercially available.

To date, all studies of the DEKA Arm reported on subjects who utilized IMUs [1] worn on the feet as the primary control method [2]. IMUs were used in combination with pressure transducers and traditional EMG controls and switches, as needed or desired. The IMUs are mounted on a clip that is attached to the top of the shoe and utilize gyroscopes and micro electromechanical systems (MEMS) accelerometers to sense small movements of the foot/ankle in space. The user commands motion of the prosthesis by tilting their foot (and the IMU) in various directions. However, foot controls cannot be used for persons who are missing both lower limbs or who have lower limb paralysis or serious impairment. Furthermore, for safety reasons the IMUs are designed to automatically stop functioning during ambulation due to a “walk detect” feature which minimizes the possibility of inadvertently moving the device while walking. The DEKA Arm also has a “standby” feature, which enables the user to disable the controls without powering off. This is typically operated using a pressure transducer.

There are three configuration levels of the DEKA Arm; radial configuration (RC) for persons with transradial amputation (TR); the humeral configuration (HC) for persons with transhumeral amputation (TH); and the shoulder configuration (SC) for persons with a short residual limb, a TH amputation, and shoulder disarticulation or forequarter amputation. [2] All configurations use control inputs for the hand and wrist including: grip selection (to choose between six different grip patterns) wrist flexion and extension, wrist pronation and supination, and opening and closing of the hand. All configurations of the device have a wrist display which shows the grip pattern that is currently selected as well as the mode of operation. The HC DEKA Arm uses control inputs for the movements of elbow flexion and extension and humeral internal and external rotation; the SC DEKA Arm uses a unique control strategy called Endpoint control [3]. Both the RC and HC devices utilize an external battery pack, typically worn on a belt secure around the waist. However, the HC device also has an internal battery and because of this the HC device may be disconnected from the external battery and operated without it for brief periods. Internal battery life varies depending upon the amount of motor usage but typically ranges from 1-2 hours.

Regardless of the control strategy, there is substantial cognitive load required for the complex task of pre-planning and sequentially controlling multiple joints/actuators. We believe that this cognitive burden may have been increased when substituting foot movements for upper body movements. This may explain, in part, why a kinematic study reported that using the DEKA Arm was slower than using conventional myoelectric devices for TR amputees [4] and that these subjects, despite training, did not take full advantage of the wrist motion available.

Recent commercial release of an EMG pattern recognition (EMG-PR) system holds promise for decreasing cognitive burden of using the DEKA Arm. In the past decade or so, there have been major advances in EMG-PR control, [5–11] culminating in the launch of the first commercial EMG pattern recognition product, CoAPT COMPLETE CONTROL™ system in 2014 (http://www.coaptengineering.com). In EMG-PR, the controller deciphers the user intent based on activation patterns of residual muscle when the user imagines and “executes” the motion of the missing limb. The controller then selects the corresponding motor and controls the speed in proportion to the sum of the EMG intensity of all monitoring signals. Compared to the conventional direct EMG control scheme, EMG-PR does not rely on independent muscle contraction. Specifically, EMG-PR utilizes the activation patterns of muscles, chosen by the individual patient, that are mapped to prosthesis movements through the process of calibration.

We anticipated, based on results reported in several recent studies, that an EMG-PR control scheme may enable users to control multiple DOFs of the DEKA Arm more efficiently, [9, 12, 13] be easier to learn to use, [14] and be less cognitively demanding,[15] as compared to IMU foot controls in combination with direct EMG control. However, this control method was new and unstudied. Thus, the purposes of our study were to: (1) utilize EMG-PR to control the DEKA Arm; and (2) describe participants’ experience of controlling the DEKA Arm using this control strategy.

## Materials and methods

### Study design

The study of EMG-PR was conducted as part of the VA Home Study of an Advanced Upper Limb Prosthesis (Home Study), a quasi-experimental study that used a time series design. The research was approved by the Institutional Review Boards of the Providence VA Medical Center, the James Haley VA, the VA NY Health Harbor System, and the Center for the Intrepid at Brooke Army Medical Center. The study consisted of in-laboratory training (Part A); and up to 12 weeks of home use with in-person re-evaluations at study sites, at monthly intervals (Part B).

### Coapt – DEKA arm integration

The study team worked with Coapt LLC (Coapt LLC, Chicago, IL) and DEKA Integrated Solutions (DEKA Integrated Solutions Corp, Manchester, NH) to create an integration of Coapt EMG-PR system (referred to as EMG-PR or Coapt in this paper) and the DEKA Arm for this study. Two prototypes of the EMG-PR controls were utilized in this study: Prototype 1 and Prototype 2. Major differences between prototypes are highlighted in Table 1.

**Table 1:** EMG-PR controls to DEKA integration: Comparison of prototypes 1 and 2.

Features	Prototype 1	Prototype 2
**Computer operating system**	Windows XP/7 or greater.	Windows 7/10.
**Connection to the DEKA Arm and power**	2 ACI’s and the EMG-PR interface cable.	DEKA-CAN bus connection.
**Power**	Powered through the DEKA Arm.	Powered through the DEKA Arm.
**Arm Control Interface (ACI)**	Externally mounted ACIs are mandatory.	Externally mounted ACIs used to add tactors, IMU or pressure transducers (as needed).
**Tactor**	The tactor is connected to the ACI.	The tactor is connected to the direct wiring setup.
**Degrees of freedom (DOF)**	A maximum of 4 antagonistic DOF pairs (or functions) can be controlled without switching. Mode switching can be used to increase to enable control of 4 DOF.	A maximum of 5 antagonistic DOF pairs and 1 bi-directional function (grip toggle*) can be controlled without mode switching.
**Standby control**	Controlled by pressure transducer.	Controlled by pressure transducer.
**Mode switching**	For HC configurations, a pressure transducer was used to control mode switching.	None needed.

* Grip toggle is a function that allows the user to sequentially toggle through the 6 grip patterns to select the desired grip pattern.

**Fig 1:**
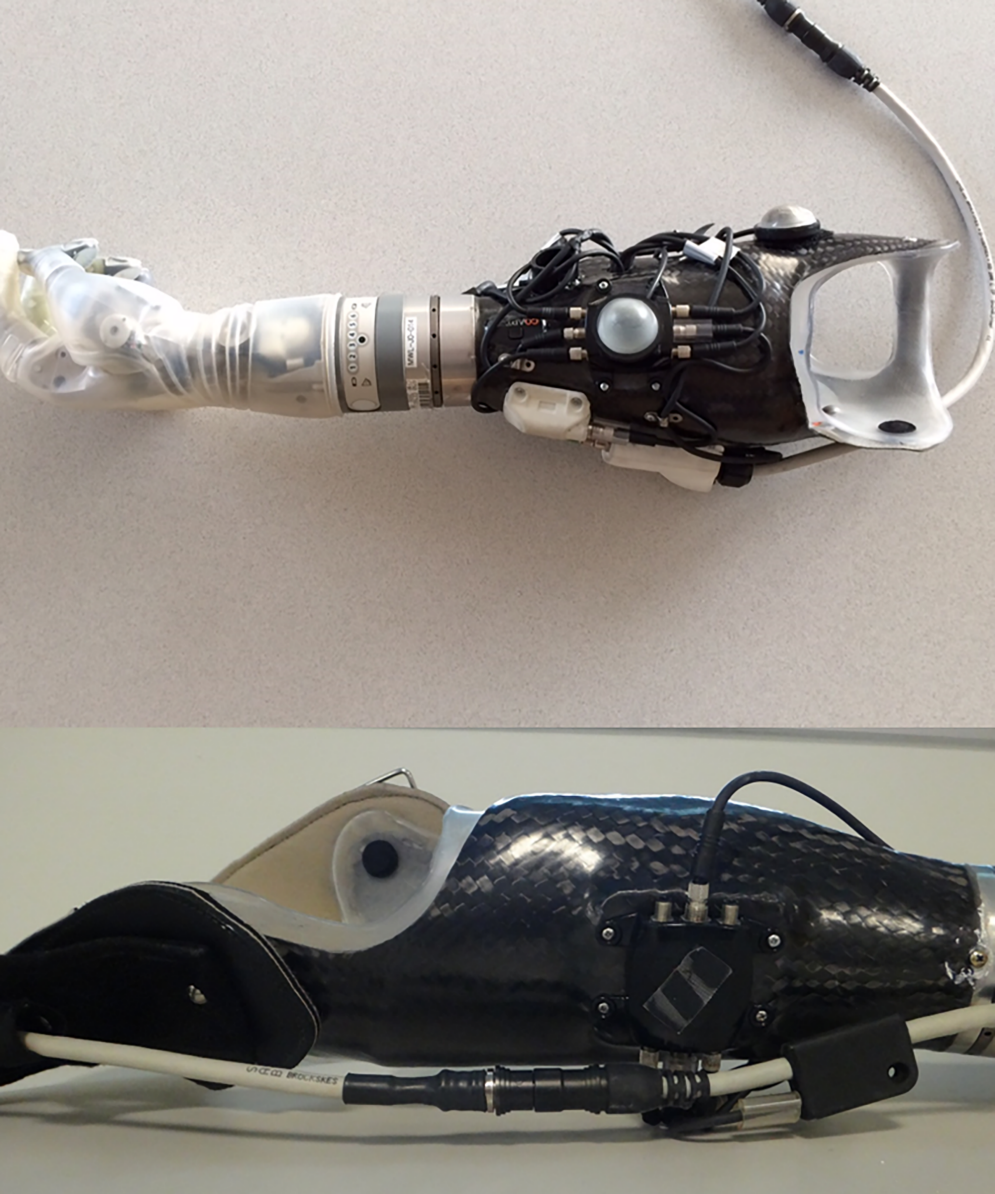
Prototype 1 RC socket.

Prototype 1 could be configured to utilize up to 8 distinct movement patterns and allowed mode switching to expand the number of DOF and functions that could be controlled. Thus, Prototype 1 with mode switching could control up to 4 DOFs in the DEKA Arm (3 for an RC device and 4 for an HC device). Prototype 1’s input for mode switching could be used to toggle between hand grip patterns to select the desired grip (aka grip selection) in both the RC and HC configurations. Mode switching was a selection function only, it did not involve movement of the prosthesis joints. In Prototype 1 the wires for the 8 dome electrode pairs, and 1 reference electrode were connected to the Coapt control system processor and then to the DEKA Arm through an multi-connection interface cable (i.e. mating cable), which connected to DEKA Arm Control Interface (ACI) units mounted on the external socket (Fig 1). The DEKA Arm tactor is an output device which utilized vibrations that provided tactile and auditory (i.e. the user can hear the vibrations) feedback to the user about grip force and mode switching. The tactor as well as any pressure transducers used for control of grip selection or mode switching, were also connected to the DEKA Arm through an ACI.

RC DEKA Arm and socket with EMG-PR prototype 1 controls. Key device components shown in this picture include: the pressure transducer, wrist display, externally mounted ACI, ACI wiring, tactor, and power cable.

Prototype 2 could be configured to operate up to 6 functions by using up to12 unique patterns of muscular recruitment (not shown). Thus, Prototype 2 did not require mode switching for grip selection or humeral rotation control. Prototype 2 interfaced with the DEKA Arm directly through use of a single CAN Bus connection. As in Prototype 1, tactors and pressure transducers were connected to an externally mounted ACI. In this study, all participants, regardless of Prototype, opted to use a pressure transducer for control of standby mode of the device.

Calibration, which is the process of training the control system to recognize the patterns of muscle signals for specific prosthesis action. During calibration, the control system records the pattern of muscle signals that are used for the movement, and utilizes this information to control the prosthesis. Calibration could be done using the software interface, which displayed a virtual prosthesis, or through prosthesis guided calibration [16] with the DEKA Arm enabled. In prosthesis guided calibration, the prosthesis moves through each movement in sequence, and the user produces the corresponding muscular patterns for each movement. [17] Both Prototypes used the calibration process available in the commercially available EMG-PR system; albeit with a greater number of DOFs and functions that could be calibrated. Study participants were introduced to the calibration process using the virtual prosthesis in the software environment and then, once they were familiar with the process, advanced to calibrating using the prosthesis guided calibration.

Study prosthetists and therapists were oriented to the system and EMG-PR and instructed in EMG-PR training principles, provided with an operations manual and reading list. The training was repeated and the operations were manually updated when Prototype 2 became available. Study staff also had telephone and on-site consultation from Coapt specialists and from DEKA as needed to address technical issues.

### Subjects

A convenience sample of persons with unilateral or bilateral TR or TH amputation who met eligibility criteria was included. Eligible participants for Part A were at least 18 years old, with no health conditions limiting study participation. At the conclusion of Part A, the Principal Investigator and the local study staff determined whether the subject met eligibility criteria for Part B. Included Part B participants had, in the judgment of the Principal Investigator and study therapist, at least fair functional use of the DEKA Arm, demonstrated safety awareness and sound judgement, and possessed the ability to troubleshoot minor technical issues.

### Data collection

At baseline, participants reported the type of prosthesis they used as well as the number of years that they had been using a prosthesis. Skillfulness of personal prosthesis use was measured using the University of New Brunswick Test of Prosthetic Function for Unilateral Amputees. Qualitative data were collected through open-ended and structured survey questions and semi-structured interviews (SGIs) at the End of Parts A and B (Appendix A in S1 File). The SGIs were audio-taped. The audio files were transcribed verbatim. Audio and video recordings were made of study training and testing sessions, when feasible.

Semi-structured interviews were administered by study staff and allowed for additional probing to elicit further information, and/or clarify responses. Surveys were administered by pencil and paper at the end of Parts A and B. Participants were asked about their impressions of the DEKA Arm, if they wanted to receive a DEKA Arm in the future, to rate their skill level with the experimental system, and to rate comfort and weight of the prosthesis. The subset of participants who were prosthesis users at baseline were asked about activities they could do and preferred to do with the DEKA Arm and not their personal device(s) and the reverse. Furthermore, the Part A survey solicited participant’s impression of whether they had enough training.

SGI questions (Appendix A in S1 File) solicited participants’ opinions on their training experience, the DEKA Arm and its components, the EMG-PR control and their function using the experimental system. Participants were asked to compare the experimental system to their personal prosthetic devices and control schemes, Participants were also asked to comment on the need for repair and service of the system. At the End of Part A (EOA), participants were asked if they thought that they were ready to utilize the system at home. At the End of Part B (EOB), participants were asked to comment on their home use experience.

### Data analyses

We used a qualitative case series design[18] with a constant comparative approach to identify common categories of experience using the EMG-PR controls. Data sources described above were used in conjunction with individual case summaries developed by study analysts in the qualitative analysis. Case summaries provided important contextual information, gleaned from communications with the study staff and/or videotaped training sessions, not necessarily available through other source materials.

**Fig 2:**
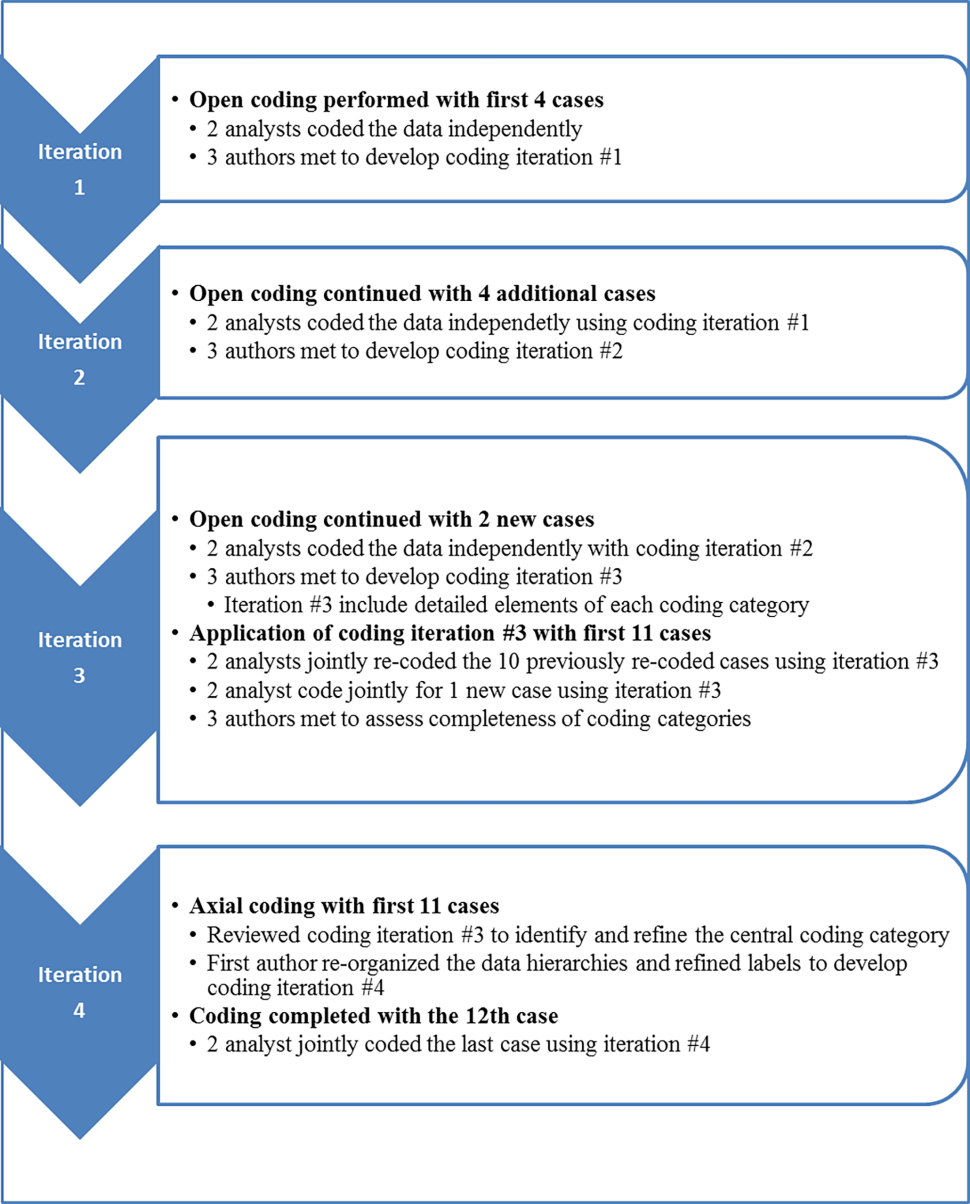
Summary of coding process.

Development of the coding structure was an iterative process which occurred during and after data collection. Data analysis was facilitated by the use of NViVO software which was used to manually code and organize text data. Fig 2 summarizes the coding process. Coding began when two qualitative analysts independently coded the data from four participants to identify major categories in the data. The analysts then met with the first author (LR) to discuss the commonalities and differences in the major categories and then write preliminary definitions of each category. This resulted in coding iteration#1. These definitions were then deployed by the analysts as they coded data from four additional participants. The process was repeated, and the coding categories and content compared, resulting in further refinement of definitions, reorganization of several categories and the addition of new categories (coding iteration #2).

Coding iteration #2 included many sub-categories which were generated to appreciate the scope and content of each category. The revised coding schema was then utilized to code 2 additional cases and the constant comparative process repeated again and coding iteration #3 was generated. The analysts re-coded the 4 original cases using coding iteration #3, constantly comparing exemplars within each category to insure appropriate classification. These results were discussed with LR, and together the team determined that no new categories were identified in the data and that saturation of coding categories had been reached. The two analysts then met to apply the coding scheme (iteration #3) to all 10 cases, resolving any differences of interpretation together during the coding session. Thereafter one additional case, the last subject who had completed Part B, was jointly coded by both analysts utilizing coding iteration #3. Summary reports of the content of each coding category were written by the analysts and reviewed by the first author who further organized the categories by identifying a central, or primary category, the major categories of user experience, factors impacting experience, and broader contextual factors (coding iteration #4). Coding iteration #4 was shared with the qualitative analysts who agreed that organization made sense and that all data was represented in coding iteration #4. Finally, the data for the last subject in the study, who did not complete Part A, was coded by both analysts using coding iteration #4. Table 2 shows the major categories and subcategories of each of the coding iterations.

**Table 2:** Major categories and subcategories of coding iterations: Subject experience with EMG-PR and DEKA.

	Coding iteration#1	Coding iteration#2	Coding iteration#3	Coding iteration#4
Major category	Other factors coloring study participation	Other factors coloring study participation	N/A	Experience of EMG-PR control of the DEKA Arm
Subcategories	N/A*	N/A	N/A*	• Desirability • Complexity • Calibration • Function
Major category	Overall desirability of system	Overall desirability of system	Overall desirability	Factors affecting experience
Subcategories	N/A*	N/A*	• Coapt Appearance • Desirability of PR for current/other system control • Desirability of the DEKA Arm • Tradeoffs related to Coapt Controls	• Training and acclimation • Fatigue • Prosthesis design • Technical issues
Major category	Weight as it affects Coapt controls	Weight as it affects Coapt controls	Contextual Factors	Contextual factors
Subcategories	N/A*	• General impact of weight • Impact of weight on electrode contact	• Other factors affecting study participation • Factors affecting credibility of subject’s perspective or responses	• Personal factors • Environmental factors
Major category	Malfunctions, repairs and service related to Coapt	Malfunctions, repairs and service related to Coapt	Malfunctions, repairs and service related to Coapt	
Subcategories	N/A*	• Other malfunctions, technical problems, repairs, faults • Reliability and consistency • Control adjustments	• Other malfunctions, technical problems, repairs, faults, reliability and consistency • Control adjustments	
Major category	Ease of learning Coapt controls	Ease of learning Coapt controls	Coapt controls	
Subcategories	N/A*	• Learning to calibrate • Learning to operate multiple controls • Muscular deconditioning • Complexity • Other factors affecting learning • Training protocol and strategies • Frustration level	• Impact of weight: Part A;Part B • Calibration: Part A; Part B • Fatigue: Part A; Part B • Complexity: Part A; Part B • Training protocol and strategies • Other Factors: Part A; Part B • Acclimation: Part A; Part B	
Major category	Function	Function	Function	
Subcategories	N/A*	• Coapt impact on function • Design of DEKA Arm	• Coapt impact on function • Design of DEKA Arm	
Major category	Ease of Use of Coapt Control of prosthesis (once controls were learned)	Ease of Use of Coapt Control of prosthesis (once controls were learned)		
Subcategories	N/A*	• Part A • Acclimation • Intuitiveness • Fatigue • Complexity • Calibration • Other factors affecting use Part B.		
Major category	Appearance related to Coapt	Appearance related to Coapt		
Subcategories	N/A	N/A		
Major category	Tradeoff related to Coapt control	Tradeoff related to Coapt control		
Subcategories	N/A	N/A		
Major category	N/A	Staff Proficiency		
Subcategories	N/A	N/A		

*N/A (not applicable)

## Results

Characteristics of the study sample by amputation level are shown in Table 3. Characteristics of individual participants (identified by pseudonyms) are shown in Table 4. Briefly, the sample consisted of 12 persons with upper limb amputation (TR = 10; TH = 2; males = 10). Both participants with TH amputation had undergone targeted muscle reinnervation (TMR), a surgical procedure in which severed nerves that previously controlled arm/hand function are transferred onto targeted de-innervated muscles on the residual limb. This procedure allows the user to use physiologically appropriate motor control signals when operating prosthetic myoelectric controls. [19] Eleven participants (92%) were users of a personal prosthesis at baseline; 4 (33%) had prior experience controlling the DEKA Arm using foot controls; and 2 (17%) had prior experience using EMG-PR with another prosthesis. Three participants (all TR) used only Prototype 1 of EMG-PR and DEKA integration, 2 participants utilized both prototypes (1TR, 1 TH). Seven participants (6 TR, 1TH) used only Prototype 2. Eleven participants completed all Part A activities (9 TR, 2 TH); 8 participants began Part B and 7 completed (5 TR, 2 TH). One participant was lost to follow-up and did not complete a SGI or structured surveys in Part A. Because of this, we used mid-study data and selected transcripts from training videos in his analysis.

**Table 3:** Characteristics of participants by amputation level.

	TR	TH	Total
	N(%)	N(%)	N(%)
**Began training**	10 (100%)	2(100%)	12(100%)
**Sex**			
Male	8(80%)	2(100%)	10 (83%)
Female	2(20%)	0(0%)	2 (17%)
**EMG-PR - DEKA integration**			
Prototype 1	3 (30%)	0	3(25%)
Prototype 2	6 (60%)	0	6(50%)
Both prototypes	1(10%)	1 (100%)	3(25%)
Prosthesis user at time of enrollment			
Yes	10(100%)	1(50%)	11(92%)
No	0	1(50%)	1(8%)
Tested with a prosthesis at baseline			
Yes	8 (80)	1 (50.0)	9 (75)
No	2 (20)	1 (50.0)	3 (25)
Mean Age ± SD	46±17.14	32± 5.65	44 ±16.56
**Prior DEKA Experience**			
Home study participant w/IMUs	2 (20%)	0(0%)	2 (17%)
Optimization study participant	1 (10%)	1(50%)	2 (17%)
**Experienced EMG-PR user**	1 (10%)	1(50%)	2 (17%)
**TMR**	0	2(100%)	2(100%)
**Study Status**			
Completed A	9 (90%)	2 (100%)	11 (100)
Deemed ineligible for B	2 (20%)	0	2(17%)
Did not continue to B	2(20%)	0	2((17%)
Began B	6 (60%)	2 (100%)	8 (75%)
Completed B	5(50%)	2 (100%)	7 (58%)
Completed but discontinued using the prosthesis during Part B	2(20%)	1 (50%)	3(34%)

**Table 4:** Characteristics of participants.

Name*	Amp level	Congenital Etiology	Gender	Prosthesis user	Months User	Baseline Primary device	EMG-PR familiarity	UNB Skill	DEKA familiarity	Hx of TMR	Finished Part A	Part A Training Hours	Extra training before Part B	Finished Part B
Tom	TR	No	Male	Yes	360	N/A	No	N/A	Yes	No	Yes	40	0	No
Mary	TR	Yes	Female	Yes	320	Myoelectric	No	3.7	Yes	No	Yes	30	N/A	No
Adam	TR	No	Male	Yes	25	Myoelectric	No	2.8	No	No	Yes	16	0	No
Martin	TR	No	Male	Yes	360	N/A	No	N/A	Yes	No	Yes	14	N/A	Yes
Suzie	TR	No	Female	Yes	5	Myoelectric	No	2.1	No	No	Yes	16	0	Yes
Jason	TR	No	Male	Yes	1	Body powered	No	3.0	No	No	No	24	N/A	No
James	TR	No	Male	Yes	18	Myoelectric	No	2.9	No	No	Yes	22	N/A	No
Charles	TR	No	Male	Yes	30	Myoelectric	No	3.1	No	No	Yes	16	N/A	Yes
George	TR	No	Male	Yes	18	Myoelectric	No	4.0	No	No	Yes	16	0	Yes
Matt	TR	No	Male	Yes	480	Myoelectric	Yes	2.0	No	No	Yes	28	0	Yes
John	TH	No	Male	Yes	70	Myoelectric	Yes	3.5	No	Yes	Yes	30	0	Yes
David	TH	No	Male	No	N/A	N/A	No	N/A	Yes	Yes	Yes	20	Yes	Yes

*Pseudonym

N/A = data not available

UNB = University of New Brunswick Test of Prosthetic Function for Unilateral Amputees. The UNB was administered to participants using their personal prosthesis at baseline. UNB activities are rated on a 5-point scale of 0-4 (0 = prosthesis not used; 4 = Active use of terminal device is quick, skilled and smooth. Grasp is consistently maintained). Average scores are reported, with higher scores indicating better performance.

Two Part B completers (1 TH, 1 TR) discontinued using the DEKA Arm at home and another (TR) chose to use the device minimally during much of Part B. The participant with TH amputation stopped using the device for the last 9 weeks; one TR participant stopped using it for the last 6 weeks; and another TR participant wore/used the device for only 1 hour during the last 3 weeks of Part B. In addition, the other subject with TH amputation decreased his prosthesis use during the last 8 weeks of Part B due to hot and humid weather.

Seven subjects completed Part B activities. Two completers of Part A (both TR) were deemed ineligible for Part B because they had not achieved sufficient prosthesis control within the training time completed. One had completed 20 training visits (about 40 hours) and the other chose to end study participation after 8 visits (about 16 hours), declining additional training that was offered. One Part A completer (TR) chose not to continue into Part B, stating that he was too busy to participate, however the study staff noted that he had a low frustration tolerance and did not feel comfortable wearing the device in public.

### Changes to EMG-PR settings during study participation

Six participants had modifications to their controls during Part A of the study (Table 5). The most common type of modification was changing from PR control of grip selection to the use of a pressure transducer. Although not shown in Table 5, the majority of participants altered or refined the muscular recruitment strategies that they used for specific movement controls. Control of grip selection was used successfully for 3 of 4 participants who used Prototype 1. One participant had difficulty controlling 3 DOF using PR and was provided with an IMU to control the pronation/supination movement.

**Table 5:** Synopsis of prototype use, control configuration, major changes to controls configuration, repair episodes, training hours, prior study participation and controls experience.

			Part A				Part B							
Name*			Configuration/change^			Repair Episodes	Participation		Configuration/change^					Repair Episodes
	**Any change**	**Prototype**	**Grip select**	**Wrist rotation**	**Humeral rotation**	**Number**	**Part B?**	**Weeks**	**Any change**	**Prototype**	**Grip select**	**Wrist rotation**	**Humeral rotation**	**Number**
**Tom**	**Y**	**1**	**PT**	**PR/IMU**	**NA**	**0**	**N**	**NA**	**NA**	**NA**	**NA**	**NA**	**NA**	**NA**
**Mary**	**Y**	**2**	**PT/PR/PT**	**PR**	**NA**	**3**	**Y**	**6**	**N**	**2**	**PT**	**PR**	**NA**	**1**
**Adam**	**Y**	**2**	**PR/ PT**	**PR**	**NA-**	**1**	**N**	**NA**	**NA**	**NA**	**NA**	**NA**	**NA**	
**Martin**	**N**	**2**	**PT**	**PR**	**NA**	**3**	**Y**	**12**	**N**	**2**	**PT**	**PR**	**NA**	**9**
**Suzie**	**N**	**2**	**PT**	**PR**	**NA**	**3**	**Y**	**12**	**N**	**2**	**PT**	**PR**	**NA**	**3**
**Jason**	**N**	**2**	**PT**	**PR**	**NA**	**4**	**N**	**NA**	**NA**	**NA**	**NA**	**NA**	**NA**	**NA**
**James**	**N**	**1**	**PR/PT**	**PR**	**NA**	**1**	**N**	**NA**	**NA**	**NA**	**NA**	**NA**	**NA**	**NA**
**Charles**	**N**	**1**	**PR**	**PR**	**NA-**	**1**	**Y**	**12**	**Y**	**1**	**PR**	**PR**	**PR**	**0**
**George**	**Y**	**1**	**PR**	**PR**	**NA**	**2**	**Y**	**12**	**Y**	**1/2**	**PR**	**PR**	**NA**	**8**
**Matt**	**N**	**1**	**PR**	**PR**	**NA**	**2**	**Y**	**12**	**N**	**1**	**PR**	**PR**	**NA**	**8**
**John**	**Y**	**2**	**PR/PT**	**PR**	**PR**	**3**	**Y**	**12**	**N**	**2**	**PT**	**PR**	**PR**	**1**
**David**	**Y**	**1**	**PT**	**PR**	**PR**	**2**	**Y**	**12**	**Y**	**1/2**	**PT**	**PR**	**PR**	**1**

PR = controls configured on EMG-PR; PT = controls configured on pressure transducer; NA = not applicable

^if no change in configuration was made only the initial configuration is shown

*Pseudonym

Although it was possible to control grip selection using the EMG-PR controls of Prototype 2 system, by toggling through the 6 grip patterns to select the desired grip, all four subjects who tried doing so encountered difficulties with this function. Three transitioned to using a pressure transducer for grip selection. When these types of changes occurred, the subject had additional practice (training hours) using the new control schemes before final testing. Given the difficulty in controlling grip selection through EMG-PR, and the frustration for study staff and subjects, subsequent subjects using Prototype 2 had grip selection set-up initially with pressure transducer control.

One participant (TH) transitioned from Prototype 1 to Prototype 2 as soon as it became available, which occurred after the completion of Part A training and before the initiation of Part B. Given the timing, the participant returned to the study site and had the control system updated. At that time, he had 2.5 hours of training delivered over a two day period. At the end of the two days, the OT reported that he was able to operate all controls well and the subject indicated that he felt ready to take the system home.

A second participant (TR) also transitioned from Prototype 1 to Prototype 2 prior to the start of Part B and after the EOA. The OT and prosthetist determined that this subject required no additional training hours given that the control scheme for Prototype 1 and 2 were set up identically.

### Overview of qualitative findings

**Fig 3:**
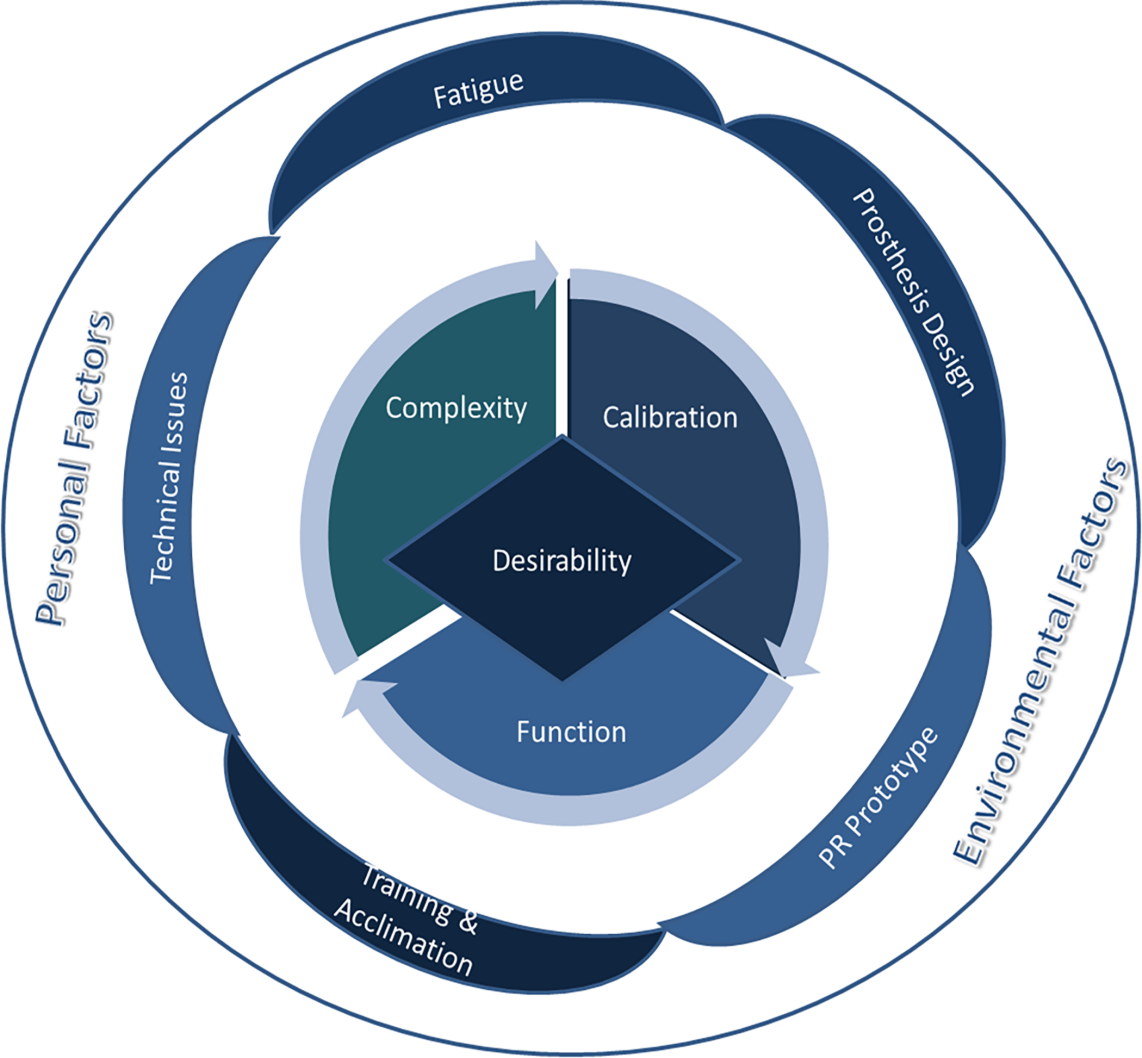
Theoretical model resulting from the analysis.

The overall organization of the coding categories are displayed in Fig 3 and the content of each major category is described briefly in Table 6. The section below contains descriptions of the findings, organized using coding iteration #4, with illustrative examples from the rich text data.

**Table 6:** Final coding scheme.

**Experience of EMG-PR Control of the DEKA Arm**	
Desirability	Comments about the overall desirability of the EMG-PR controlled DEKA Arm.
Complexity	Comments on the complexity of learning to use and using the controls. Comments include, but are not limited to the time and training required to master controls, the intuitiveness or naturalness of controls, the impact of complexity and the perceived interaction of complexity and control consistency
Calibration	Comments about the process of calibration.
Function	Comments about skill, safety, or prosthetic preference in task performance (abilities) when using system and comparison to function of other prosthetic systems.
**Factors affecting experience**	
Training and acclimation	Comments about sufficiency of training and training strategies that may have facilitated learning, and statements indicating how well participants felt that they had adjusted to using the system.
Fatigue	Comments describing the fatiguing effects of using the controls and the impact of fatigue on prosthesis operation.
Prosthesis design	Comments about the design of the DEKA Arm and its components such as the pressure transducer, weight, speed, functionality, ease of donning, battery pack, cables, size, shape, durability, and/or battery life.
Technical issues	Comments about malfunctions, technical problems, breakages, repair, service and/or adjustments during the study.
Changes to controls	Comments about changes to controls or controls prototype.
**Contextual factors**	
Personal factors	Personal factors that may have affected learning and using controls such as prior controls experience, pain or discomfort, ability to recruit residual musculature, excessive sweating, residual limb volume fluctuations, health problems, frustration tolerance, availability and willingness to participate in training, ability to retain information and credibility of subjects’ self-reported data.
Environmental factors	Contextual factors in the environment including staff proficiency, hot weather, other weather events, rapport with staff.

Fig 3 shows the theoretical model resulting from our analysis. The desirability of EMG-PR and the DEKA Arm system is shaped by the user experience of the system’s complexity, calibration and function, as shown in the central circle of the figure. These aspects are largely explained by the categories shown in the inner ring of the figure: technical issues, fatigue, prosthesis design, EMG-PR prototype, training and acclimation. Finally, personality and environmental factors, as shown in the figure’s outer ring are contextual factors that influence the entire model.

### Experience of EMG-PR control of the DEKA Arm

#### Desirability of the system

Part B participants had mixed views on the overall desirability and usability of the system. Two subjects (both TR) stated that they wanted an EMG-PR controlled DEKA Arm, while two others (1 TH, 1 TR) stated that they might want one in the future. Three subjects (1 TH, 2 TR) said they did not want one. At EOB, the 6 participants who were prosthesis users at baseline were asked to compare the controls of the DEKA Arm to the controls in their personal prosthesis. Four (66%) indicated that they preferred their own controls, and 2 (33%) did not express a preference for either control type. Several subjects, including both users with TH amputation and a history of TMR, made very positive comments about EMG-PR independent of the interface with the DEKA Arm.

“*I think that is potentially one of the most advancing systems I’ve seen in…forever*. *I mean*, *that’s definitely I think the next step from like*, *where the TMR is…Going from the*, *you know*, *basic two bicep/tricep muscles to the four with the TMR*, *and then to the Coapt system with the TMR*.” (David, Part B)

Some subjects with TR level amputation recognized that the system that they used was an early prototype and spoke about the potential of using EMG-PR with the DEKA Arm “if the fundamental problems can be addressed.” One believed that EMG-PR could eventually be the optimal means of controlling the multiple DOFs of the DEKA prosthesis:

*“…I think that once all of the problems are worked out and the integration is completely done*, *figuring out how the 2 technologies work together*, *I think the Coapt system or pattern recognition system in general will be the best way to move the hand*, *because there are so many degrees of freedom and it allows for such intuitive control with that many degrees of freedom*.” (Mary, Part A).

Subjects mentioned several features of the DEKA Arm which dampened or added to their enthusiasm for the device. Ten of eleven subjects who completed Part A and 5 of the 7 who completed Part B commented negatively on the weight or bulk of the DEKA Arm system, remarking that it was “too heavy,” that the “weight is very frustrating”, or the device is “bulky” or “cumbersome.” The size of the prosthetic socket with the externally mounted componentry, and the shape of the hand itself made it difficult for some subjects to wear their usual clothing. Subjects made negative comments about the external battery pack and wiring. One subject said there was “too much hanging off it.” Another remarked,

“*It didn’t work near well enough to warrant dealing with the crap hanging off of it*, *especially the battery*, *belt and cord*. *Try taking off a coat and using a public restroom wearing that get up*.” (George, Part B)

Participants commented positively on the powered wrist movements of the DEKA Arm. One subject with TR amputation explained that while the need for repeated repairs had a “large effect” on her interest in obtaining a DEKA Arm in the future, the benefit of having a wrist that was able to able to “rotate, flex and extend” was “definitely worth it.”

One subject (TR),who called the DEKA Arm “not ready for prime time yet,” reported that he used the Arm only for 1 hour at home during the last 3 weeks of Part B, explaining that the DEKA Arm, despite its multiple DOFs, made him less “productive:”

A third subject (TR) summed up the tradeoffs between functionality of the system and need for frequent repair:

*“……I could do chores around the house*, *mowing the lawn and stuff*, *that was definitely a bonus*, *but every time I mowed the lawn the hand would break then I’d get frustrated*, *because then I wouldn’t wear it as long because it was broken so I couldn’t wear it*.*”* (Martin, Part B)

A fourth subject with bilateral lower limb amputation explained his preference for his own simpler terminal device:

*“I just left it (DEKA Arm) there and I was like*, *’I’m putting my own arm on*.*’ It’s faster*… *easier…After a while you’re like*, *‘this just takes too long to do stuff with this hand*.*’ And I could just like throw mine on*, *throw a Greifer on it and call it a day*.*”* (Charles, Part B)

This subject, who sometimes utilized a wheelchair and sometimes walked with a cane, also reported that the external battery pack rubbed against the side of the chair, the device was complicated to wear when seated in the wheelchair, and the wrist bent under the weight of his arm when using a cane.

#### Complexity and intuitiveness of controlling DEKA Arm with EMG-PR

All subjects who completed Part A commented on the complexity of using the EMG-PR controls to operate the DEKA Arm saying that it was “more complicated”, “harder”, “more difficult”, and “a more tedious process” compared to other prosthetic control systems that they had used. Because of the powered DOFs of the DEKA Arm, subjects required more control inputs; sometimes two or three times more than their personal devices. Subjects needed to remember, and then replicate, distinct patterns of muscular contractions for each control. Those who were already using myoelectric controls needed to use new muscular patterns that may have differed from their current control systems.

During Part A one subject who was accustomed to using two-site myoelectric control remarked that learning to use the new controls was, “like a re-wiring of the brain and the muscles.” This process, he said, at EOA, was “exhausting” and “too much,” and he thought that it would require “a lot of training” to become proficient. At EOB, the subject stated that he preferred the control system of his current prosthesis due to “the simplicity,” explaining that using his controls was “easy,” in part because he was familiar with them and that he did not need to calibrate them.

Another subject with TR amputation compared the EMG-PR controls to his current dual site myoelectric controls, noting, “it’s a lot harder to use” because “it’s got more complexity.” The complexity, he reported at EOA, “sometimes gets in the way”, causing inadvertent movement. He explained,

“*So it can do things*, *but if I’m focusing on something and*, *this comes to not having worn it enough*, *and not having used it enough*, *but*, *you know*, *I will inadvertently open it*, *or I’ll inadvertently flex or extend the wrist when I’m trying to do something*.*”* (George, Part A)

At EOB, this subject reported that he continued to have difficulty discriminating between all of the control inputs to utilize the system with the DEKA Arm. However, he was very positive about the potential for EMG-PR as a system of control in general, and believed that it would have been easier to use with a prosthesis with fewer DOFs. He remarked,

*“*…*slap the Coapt and Michelangelo on me tomorrow*, *and it’ll go off like gangbusters*” (George, Part B).

This subject was one of two who thought that it would have been easier to master the PR control system if they had been exposed to it at the time of amputation, prior to any other prosthetic training. This subject explained that initially his sense of his phantom limb, which he engaged in using the controls, was stronger.

*“…my hand was amputated closing in on three years ago… I actually was able to work with it*. *I could feel each individual left finger*. *I could do things*, *I could move each finger independently*, *‘cause I still had sensations in them*, *but after two years plus of using a myo*, *a standard myo with just two sites*, *you lose all those sensations*. *And so the ability to discriminate between rotating the wrist*, *flexing*, *or extending and flexing the wrist*, *and opening and closing the hand - that gets much more difficult than it would have been for me two years ago*.*”* (George, Part A)

Similarly, another TR amputee remarked that he thought it would have become “second nature” to use the controls if he had trained with the system when he was a “brand new amputee....“fresh out the hospital”.

The complexity of the control system was even greater for those using an HC DEKA Arm. These subjects needed to operate 4 more controls than those operating an RC Arm. One subject commented:

*“…it takes a lot more thought and a lot more training I feel*, *to*, *and not just like strength training and stuff*, *but just thinking of what muscles or what movements you want to make”* (David, Part A)

This subject chose to discontinue using the DEKA Arm during Part B, soon after 2 additional distinct controls were added during the system upgrade to Prototype 2. He explained that the additional complexity discouraged him from using the device, leading to abandonment of home use, commenting:

*“…doing the amount of movements that we do already with this (Prototype 1) is more than enough*. *I mean*, *we’ve got like six different functions that I’m having to do…and adding two more into it*, *it changes everything*.*”* (David, Part B)

The second HC user, who had prior experience with an EMG-PR system, said that the study experience was, “kind of humbling because I thought I was better at pattern recognition than what I am.” He remarked that he “enjoyed” the process of using EMG PR control because he could “drive the Arm” with his phantom limb, and that it felt “more in-tuned” to him. However, he noted that his current prosthesis only had three functions, and that the DEKA Arm had five functions. At EOB, although he indicated that he had fully acclimated to the EMG-PR controls, he stated:

*“…this arm is very mentally taxing*, *with all the patterns and everything like that*, *to use it to get it to work and everything like that*. *It is super*, *it takes a lot of concentration……so it took a while to figure out how to isolate all those patterns*, *‘cause you’ve got to figure you’re doing two for humeral rotation*, *you have two for elbow*, *so every movement you have two patterns for*. *You know you have the movement and then you have the opposite movement*, *so there are 11 different patterns or something…”* (John, Part B)

Another subject, a TR amputee who had prior experience with EMG-PR control, made similar comments about the complexity of controls due to the greater number of DOFs of the DEKA Arm. He reported that he felt that the EMG-PR control system used in this study was less intuitive than his other prosthesis control systems, and that it was “more difficult” than he anticipated it would be, and “less reliable,” leading to unintentional control activation. He commented:

*“…*… *there was a lot of pattern signals getting confused with each other – that’s the only way that I can put it…You try to do one thing and something else happens*, *or if you move your arm in a certain position*, *and you try to open your hand or you want to close and it opens up and you drop your utensil or whatever*.*”* (Matt, Part B)

Although most subjects spoke about the complexity of the system, 50% of the subjects who used a personal prosthesis at baseline and also completed Part B (N = 3), stated that the EMG-PR system was either more intuitive than their existing mode of control or “basically the same.” One subject who reported that it was more intuitive explained how the system adapted to him:

“*I do stuff and Coapt…Coapt records what I do*. *Coapt learns me*, *as opposed to me learning the standard myo*. *So it’s far superior and I think in the long run*, *it’s going to be definitely*, *you know*, *the go-to control mechanism…”* (George, Part B)

Three of the 4 subjects who had previously controlled the DEKA Arm with IMUs made comments comparing the two control approaches. One subject with a TR level of amputation felt the EMG-PR control was more intuitive than IMUs, but stated that she also liked the “simultaneous control” that the IMUs afforded her for wrist and hand movements, which she did not feel was possible with the EMG-PR controls.

*“… I really got used to them (IMUs) and liked the fact that they are simultaneous control*, *and I could bend my wrist while opening and closing the hand like that was a really cool part of it*. *But at the same time*, *it’s frustrating having to move my feet to move my hand*. *It’s not intuitive obviously*, *so I do like Coapt control overall because of that*, *but if it had simultaneous control it would make it better*.*”* (Mary, Part A)

Another subject with TR amputation also commented on the advantage of “not having to worry about something else” (i.e. moving one’s feet) for control, but instead depending on “the motion computing from your amputated arm.” This was the only subject in this study who, due to his inability to master the EMG-PR controls and his prior familiarity with IMU controls, was fit with an IMU for some wrist movements during training. He expressed satisfaction with the IMU for that motion and said that he preferred it to using EMG-PR control, stating. “it made it a lot easier” because “I only had to worry about two motions (with EMG-PR) instead of three.”

A subject with TH level of amputation, who was highly dissatisfied with foot controls when he participated in prior DEKA studies, continued to express this opinion at the end of this study. He felt EMG-PR was “so much more intuitive” than foot controls.

*”… it beat out the IMUs a billion to one*, *cause I hated the IMUs*. *For transhumeral*, *I wouldn’t use anything but the Coapt*.*”* (David, Part B)

He appreciated the fact that the EMG-PR controls did not involve a device mounted on his shoes and could be used when barefoot. However he observed that EMG-PR required more training and stated that for “somebody who’s never worn prosthetics before” the IMU system may be easier to learn:

*“The Coapt it takes a lot more thought and a lot more training I feel and not just strength training but just thinking of what muscles or movements you want to make*. *It’s a bit more complex but you don’t have to worry about moving your feet*. *It’s so much simpler and more complex at the same time using the Coapt as opposed to the IMUs*.*”* (David, Part A)

This subject noted that the weight of the DEKA Arm affected controlling the prosthesis with EMG-PR “a lot, more so than like the IMUs”. But the tradeoff he thought was “well worth it.”

#### Calibration

All subjects commented on the experience of controls calibration, discussing the reliability and consistency of the calibration process, the degradation of controls after extended use, and the need for and frequency of recalibration. One subject remarked,

*“we calibrated*, *it worked for a good while*. *Then something happened…the calibration wasn’t*, *adjusting to me or me adjusting to the Arm*. *It was quite difficult*. *We had to recalibrate*.*”* (Charles, Part A)

Although re-calibration typically improved prosthetic control and subjects said that they would “rather recalibrate” to obtain better controls when there was degradation, the need to calibrate at all was seen as a disadvantage as compared to current myoelectric control. In comparison, direct myoelectric control was perceived as “simple,” did not require recalibration and “will work no matter what.”

There were also occasions when the calibration process itself was not successful. One subject commented that she was not “guaranteed that it’s (the calibration process) going to work,” and thus she would need to repeat the process. All subjects spoke about strategies that they used to improve the likelihood of getting a successful calibration. Several said calibration was more successful when they wore the DEKA Arm regularly, paid greater attention during the calibration process, layered calibrations, and/or calibrated with the prosthesis weight supported as well as not supported.

One subject at EOA and 4 subjects at EOB reported that they needed to recalibrate often and would layer multiple calibrations to regain control. However, the need for and frequency of calibration varied within subjects. As one subject remarked at EOB, “sometimes I would do it two times a day and sometimes I wouldn’t have to calibrate at all.” Additionally, the number of calibration attempts needed to obtain good control was inconsistent:

“*there were times when I could calibrate once and that’s great*, *but then there were times when it was really acting up and I’d like do it six or seven times in a row before I’d get it calibrated*.*”* (Matt, Part B)

Several subjects explained that they found the calibration routine tiring, particularly because, with the prosthesis guided movement, “the Arm is moving around so intensely.” The movement of the prosthesis required the subjects to stabilize their proximal arm and shoulder using their own musculature, or sometimes using their contralateral arm or a table. One subject explained that the movement of the prosthesis on the residuum,

*“alters the way that it’s picking up the recording because it (the prosthesis on the socket) is like shaking back and forth*.*”* (Suzie, Part B)

Although users could calibrate using the computer software which displayed a virtual arm, during the study calibration using this method could only be done in laboratory with assistance from a staff member. Two subjects stated that they were able to time their muscle contractions using the virtual Arm. Another stated that he didn’t “like to watch on the laptop” and preferred to watch “my real hand” do the work, during calibration.

The two subjects who had prior experience with the EMG PR, reported that the calibration process for the EMG-PR DEKA controls was the same as they were accustomed to, but it took longer because “there’s a lot more movements.” These subjects also commented that the calibration process seemed less reliable compared to calibration with their personal prostheses.

#### Function

Several subjects made general comments describing their enhanced functionality due to ease of use of the EMG-PR system.

*“…because once I have it on I can just do things just more fluidly as opposed to pressure to open*, *pressure to closing it*.*”* (Suzie, Part B).

Most users commented on the advantages or disadvantages of the combined functionality of the controls and prosthesis. For instance, a TH user remarked that the entire DEKA Arm system with wrist flexion-extension and humeral rotation together with EMG-PR controls provided more “freedom” and “felt natural,” saying “you don’t have to angle yourself” for tasks like he needed to do with his other prosthesis. Another subject commented negatively about the wrist function, after using the prosthesis to remove a T-shirt,

“*When you get [DEKA] on all those funny angles it just gets taxing*.*” (Jason; Part A)*

One TR user described how various aspects of the entire system made him slower and less productive than when he used his existing prosthesis.

“*…it became apparent to me through the process of this that putting the DEKA Arm on made me less productive, it slowed me down…The time it takes to put it on, make sure it’s working right…I was working around it rather than using it to just do stuff*.” *(George, Part B)*

Subjects who were using a prosthesis at baseline were asked at the end of Parts A and B to comment on activities that they could do or preferred to do with the DEKA Arm compared to their own prosthesis. All prosthesis users who completed Part A (9 TR, 1 TH) used a myoelectric device at baseline. When asked to compare their own device to the DEKA Arm at EOA, 50% said that there were activities they could do with the EMG-PR controlled DEKA Arm that they could not do with their own prosthesis and 30% said that there were activities they could do with their own prosthesis that they could not do with the EMG-PR controlled DEKA Arm.

When asked whether there were activities they preferred to do with each prosthesis, 30% said that there were activities that they preferred with the DEKA Arm and 60% said that there were activities that they preferred to do with their own prosthesis. At EOB, 33% of completers said that there were activities that they could do with the DEKA Arm but not their own prosthesis, and 83% stated that there were activities that they preferred doing with their own prosthesis as compared to the DEKA Arm; 50% stated that they preferred to do everything or “just about everything” with their own prosthesis. (Table 7.)

**Table 7:** Survey items administered at end of A and end of B.

	End of A All (N = 11)		End of A+ (N = 7)	End of B (N = 7)	
	Mn (sd)		Mn (sd)	Mn (sd)	WSR P*
TAPES Satisfaction Scale	3.0 (0.6)		3.2 (0.5)	2.7 (1.1)	0.17
Self-rated skill level using DEKA Arm	2.5 (0.7)		2.7 (0.8)	2.4 (1.0)	0.75
Perception of weight of DEKA Arm	4.2 (0.8)		4.1 (0.9)	4.1 (0.7)	1.00
Rating of socket comfort	3.1 (0.7)		3.3 (0.8)	2.9 (0.7)	0.50
	**Mn (sd) Range**		**Mn (sd) Range**	**Mn (sd) Range**	WSR P*
**As of today, please rate the comfort of the socket interface with the DEKA Arm after wearing it for one hour.**					
Comfort Rating^	3.1 (0.7) 2-5		3.3 (0.8) 3-5	2.9 (0.7) 2-4	0.500
	**N (%)**	** **	**N (%)**	**N (%)**	McN P*
**Amount of training**	** **	** **	** **	** **	
Not enough	0 (0.0)		0 (0.0)	N/A	N/A
Too much	0 (0.0)		0 (0.0)	N/A	N/A
Just right	11 (100.0)		7 (100.0)	N/A	N/A
**Do you want to receive a DEKA Arm in the future?**					1.00
No	1 (9.1)		1 (14.3)	3 (42.9)	
Yes/Maybe	10 (91.0)		6 (85.7)	4 (57.1)	
**PROSTHESIS USERS ONLY**	**N = 11**	** **	**N = 6**	**N = 6**	**N = 6**
**Are there activities that you would prefer doing with the DEKA Arm rather than your current prosthetic arm?**					0.50
No	3 (30.0)		1 (16.7)	3 (50.0)	
Yes/Maybe	7 (70.0)		5 (83.3)	3 (50.0)	
**Are there activities that you would prefer doing with your current prosthetic arm rather than the DEKA Arm?**					1.00
No	3 (30.0)		2 (33.3)	1 (16.7)	
Yes/Maybe	7 (70.0)		4 (66.7)	5 (83.3)	
**Were you able to perform any new activities using the DEKA Arm that you have not been able to perform with your current prosthesis?**					0.50
No	5 (50.05)		2 (33.3)	4 (66.7)	
Yes	5 (50.05)		4 (66.7)	2 (33.3)	
**Were there any activities that you could not do with the DEKA Arm that you are able to do with your current prosthesis?**					1.00
No	7 (70.0)		5 (83.3)	4 (80.0)	
Yes	3 (30.0)		1 (16.7)	1 (20.0)	

+ End of A scores for those who also completed Part B

*Wilcoxon signed-rank and McNemar tests for categorical and dichotomous survey responses comparing subjects who completed surveys at end of Part A and end of Part B

^Comfort rating (1 = Could not tolerate needed to remove; 2 = Uncomfortable; 3 = Aware of presence, tolerable; 4 = Comfortable; 5 = Very comfortable, could wear indefinitely)

### Factors affecting user experience

#### Training and acclimation

At EOA, all subjects stated that the amount of training they received during the study “was just right” and most indicated that they expected to continue acclimating to the system with “time and repetition.” Both TH amputees commented on the importance of adequate training, remarking that acclimation “takes time.” The TH subject who discontinued usage during Part B estimated that sufficient training “would have taken three or four months” compared to the 20 hours (10 training visits) over approximately 6 weeks that he had initially received, and 2.5 additional hours after the prototype was updated. At EOB he commented,

*“People need more time to train*. *Especially if we’re going to operate it the way it’s supposed to operate*.*”* (David, Part B)

The other subject with TH amputation, who was experienced using EMG-PR controls with his personal prosthesis, said that he had fully acclimated to the device and controls. He reported that the amount of training he had received (about 30 hours over 15 visits) was adequate. He advised others learning to use the controls system to be “patient with it,” noting, “it’s a steep learning curve.”

At EOB, when asked to rate their level of acclimation to the controls system, only 2 of the 7 subjects who completed rated their acclimation as complete. “Two others stated that they were “mostly” acclimated, and 3 stated “somewhat.” Subjects who stated that they were other than completely acclimated, made comments such as: it was still “a learning curve,” and “I think I control it as good as I can for what it’s got.” Only 1 of the completing subjects rated his skill level as “excellent”, while 2 rated themselves as “good”, 3 as “fair” and 1 as “poor”. One subject who had persistent difficulty discriminating muscle muscular patterns said:

*“*,,,*if I had no other responsibilities*, *I could spend my days just practicing with DEKA and Coapt*, *I might’ve been able to get that discrimination back*. *I don’t know*, *maybe I could*, *maybe I couldn’t…”* (George, Part B)

Factors that may have interrupted the acclimation process during the home portion of the study included discomfort, pain, or skin rash which prevented wearing the prosthesis, as well as disruptions in prosthesis use due to technical problems which required repair. Three subjects, one who did not continue to Part B and 2 who did, reported that pain, numbness, or discomfort in their residual limb limited or prevented prosthesis wear. A fourth subject reported that her socket was too loose at the beginning of Part B, which limited her use; however it was adjusted during her Week 4 visit to the site. At week 8 this subject developed a persistent skin rash that prevented prosthesis wear for approximately a week. This subject who had multiple interruptions in use at home summarized the advantages of being able to “stay in a routine for wearing” the device.

*“…so it was a lot easier to use as opposed to when I didn’t wear it for a little bit and then got busy and putting it back on was like starting fresh again*.*”* (Suzie, Part B)

#### Fatigue

All subjects who participated in Part A and the majority of subjects who participated in Part B spoke about fatigue resulting from using the EMG-PR controls. Factors contributing to fatigue included; the need to perform distinct new patterns of muscular contractions, repetitions of those contractions, extended periods of prosthesis use, the weight of the DEKA Arm, and the need to recalibrate. Some subjects described a cycle wherein they became fatigued from using the controls, and then muscular fatigue led to increased difficulty reproducing the distinct muscular patterns needed for control. Their muscles were, in some cases, “working twice as much” in order to utilize the controls and this was perceived as “a lot.” One subject stated,

*“the more tired I got*, *the harder it was to give reliably consistent*, *you know*, *discriminated inputs that the Coapt was looking for*” (George, Part B).

Two transradial amputees explained how fatigue from overactive muscles led them to unintentionally activate the EMG-PR controls. One said,

“*I think what creates the inadvertent activity is muscle fatigue or muscle*, *overactive muscles*, *and that’s brought on by fatigue”* (Tom, Part A)

Another subject reported,

*“after having it [the DEKA Arm] on a couple of hours*, *especially extended use*, *and I think I found that if I was overdoing it*, *it would not operate the way I wanted it to*, *so I would do it even harder…which adds more fatigue*.*”*(Adam, Part A).

The two subjects who were experienced EMG-PR users explained that fatigue was caused by contracting unfamiliar muscular patterns to control the prostheses’ DOF.

“*because these are patterns that I’ve never made before*, *there is definitely significant muscle fatigue so I have a hard time setting that same threshold for the pattern*.*”* (John Part A).

One subject talked about the fatiguing effects of repeating contractions during the calibration layering process, and explained how this was compounded by the weight of the prosthesis. He said,

“*as I added calibrations to it*, *I was getting more tired*, *my overall calibration to a certain point seemed to like started to getting worse*…*I don’t know how much of that was me… fatigue…that I have a harder time discriminating…I was confusing Coapt*. *The weight of the DEKA influenced that to some degree*. *I just couldn’t tell you by how much*” (George, Part B).

#### Prosthesis design

The most common comments about prosthesis design affecting use of EMG-PR related to the weight of the device. Three quarters of subjects in Part A and 71% of subjects who participated in Part B commented about the impact of the prosthesis weight on their controls experience. Three subjects thought that difficulty performing muscle contractions during calibration and control was due to the prosthesis shifting weight during movement, and the resulting changes in socket pressure and surface electrode contact, which sometimes led to involuntarily muscle contraction. As one subject explained,

“*you calibrate in one position and then you start doing activities in another position and the weight absolutely changes the contact of the Coapt*…*I think it’s one of the issues why I couldn’t get control*. *Calibrating in one position start doing activities in another*” (Adam, Part A).

Another subject explained,

*“the weight could have affected with the thing*....*you are holding your muscles and they tense and you get cramps or something or you get muscle fatigue and that could send different signals to the different wrist turning and other motions and sometimes it might confuse it to where you are ”* (Martin, Part A)

The HC subject who was an experienced EMG-PR user talked about the weight shifting of the prosthesis on the residual limb, saying,

*“the weight of the Arm is actually shifted on the residual limb*, *so it makes more contact if you are reaching out to your front or to your side on different parts of that limb*, *so it is harder to set the pattern because the contact isn’t the same from the socket*. *So the thresholds for the patterns stay the same*, *but the conditions for it are not the same*. *It becomes harder to replicate because of the weight of the Arm and whatever you have in your hand*.”(John, Part B).

The prosthesis weight also caused proximal muscle fatigue:

“*I have gotten fatigued several times*, *but a lot of time I don’t think it was as much in my residual limb*, *the fatigue had to do with my shoulder carrying the weight of the DEKA*. *And occasionally there was a little bit of fatigue in my residual limb*, *but at that point*, *I just recalibrate it and it adjusted*. *Like when I’m training it*, *it adjusts for fatigue so it was never really a problem*” (Mary, Part A).

There were other issues related to the prosthesis design that influenced user experience. Although the power draw from the EMG-PR system was not expressly measured it was the user's impression that it reduced internal battery life of the DEKA arm as indicated by the statement that:

*“it sucks power like crazy*, *so I don’t think you can even last like a couple of hours*. *With the (EMG PR system)*, *that’s the biggest thing they have to address*.*”* (David, Part B)

#### Technical issues

Several subjects commented on inconsistency of controls operation. They believed that the problem stemmed from the controls system itself or integration of the controls with the prosthesis. One subject who did not wish to extend his training in Part A to become more proficient in order to participate in Part B stated:

“*I was unable to get a consistent control after many different calibrations, many hours of training. I just couldn’t get consistent control. Sometimes it would work well sometimes it would work kind of good, and sometimes it would be horrible. And so just from a consistency standpoint and reliability it was low marks*.” *(Adam, Part A)*

Factors influencing consistency of the EMG-PR controls included sweating and changes in volume of the residual limb that affected electrode contact. One subject who continued to Part B was often unable to wear the Arm during the summer months due to sweating interfering with control reliability. He explained:

*“…it was the user interface where I was sweating so much that*, *I’m just conducting too much…*.*Because the more you sweat*, *the more sensitive it becomes which makes it*, *you know*, *you’re constantly overshooting whatever you target…”*(John, Part B)

Another subject who had changes in residual limb volume reported:

*“And with Coapt like just throughout the day and your arm moving*, *or your residual limb getting bigger or smaller from heat or cold or whatever*, *affects how you control your arm or if you have to recalibrate throughout the day*. *I just feel like that changes like the consistency of control*.*”* (Mary, Part A)

A subject who had poor skill after 40 hours of training and was deemed inappropriate to continue to Part B thought that some of the problems were related to socket fit and electrode contact. This subject was also observed by staff to have difficulty with retaining information, and was only able to tolerate wearing the prosthesis for about 40 minutes at a time. The subject reported:

“*They’re (the controls) not hard to learn but what is difficult is once you learned and you calibrate it, is to transfer that information immediately. It doesn’t seem to be able to do it immediate all the time, just some of the time and I don’t know the reason for that yet. I’m suspicious of what it might be. It seems like maybe the arm swells up or shrinks or something when you put it on*.” *(Tom. Part A)*

Several subjects who participated in Part B reported similar issues of inconsistency in control operations. One stated at EOA that “there was a lot of inconsistency” which she said, “had nothing to do with me as a user.” The other stated he felt the pairing of the EMG-PR system and the DEKA Arm technologies “was kind of rushed” and he felt it would have been better to

“*have had technicians on site with it when we were actually starting to use it*, *Coapt and DEKA…because we’re putting the two systems together*.”(John, Part A)

Ninety two percent of subjects who participated in Part A and 86% who participated in Part B required at least one repair to their controls or prosthesis during the course of the study. On average subjects required 2.1 (sd 1.2) repairs during Part A, and 3.9 (sd 3.8) repairs during Part B. The range of needed repair episodes was wide, particularly during Part B. Technical issues and repairs were of the controls system, the prosthesis or a combination of both. It was often difficult for users and staff to differentiate the source of the problem.

When asked to comment on the frequency of faults or errors in controls and the need for troubleshooting, one subject said “it wasn’t acceptable.” Another subject said that he thought that the faults were “in that Coapt/DEKA integration.” One subject indicated that he thought the DEKA arm system itself was reliable, but that the control system “is not as reliable”. Another stated,

*“It is awesome when it is working well*. *However it is frustrating when it is not working”* (Jason; Part A)

One subject stated that he thought that the DEKA Arm was “too unreliable, kept breaking down” and said he did not want the DEKA Arm and the EMG-PR in the future. The same subject also said that he thought EMG-PR “could be” an effective means of controlling the DEKA Arm, despite the fact that “the wires kept coming apart” during home use. Several subjects said that their main concern about taking the DEKA Arm home was that it could “malfunction”, and that “too many things can go wrong.”

During Part B, the need to ship the prosthesis back for repairs created interruptions in study participation. One subject, who experienced 8 repair episodes during Part B, said he used the DEKA Arm “less than I expected” because it “kept breaking down”. He explained

*“it just interrupt the whole thing…that distracts you from wanting to do it because it’s like*, *“Aww*, *man*! *Here we go again*!*””* (Matt, Part B)

Another subject also commented on the impact of interruptions due to repair episodes, stating:

“*anytime you stop using something your body just gets so used to using it and your muscles*, *the memories are in there*. *It’s kind of like riding a bike*, *once you get used to it you know how to do it*.” (Suzie, Part B)

This experience was not universal however. One subject said that he was “impressed with how consistently well” the system worked. However, this was the subject who did not use or wear the device due to “sweating” for 34 days of Part B which occurred during the hot summer months.

Although subjects experienced many technical problems during the study, some were still very positive about the system. One stated,

“*when it is working*, *it’s working great*, *because it helps me do things I need assistance with*” (Martin, Part B)

#### Issues related to prototype change or grip select control

As mentioned above, two subjects had their controls system upgraded to Prototype 2. One TH amputee, who had two additional control inputs added during Part B, reported that the additional complexity of the new control inputs made him “not want to use the thing at all,” and he discontinued all use after Week 4 of Part B. This subject was also observed by staff to have a low frustration level.

The TR user who switched to Prototype 2 experienced multiple problems with the operation of grip select that required multiple visits to the site for adjustments. These problems clearly affected his perception of controls reliability and willingness to use the Arm at home.

Two subjects who used Prototype 1 with EMG-PR for grip selection throughout their protocol were generally satisfied with that form of grip control. One subject who used Prototype 1 and changed to pressure transducer during training, stated the manual mode of control was “easier” and that “pattern recognition was a huge pain” because:

*“…moving my arm in any way confuses it*, *I think*, *to where it thinks that I’m asking it to change the grip and it does when I don’t want it to*.*”* (James, Part A)

No subjects used direct grip select with Prototype 2 without experiencing technical problems. These problems may have been attributable to the experimental nature of the grip select option in these new control prototypes. Despite problems, one subject would have preferred to use EMG-PR for this function if it had been more reliable.

“*II really liked being able to use my Coapt to do the grip select. I felt like it made it a lot easier. I hate having to use the button. But I do feel like since we took the button out that the hand has been cooperating much more than it was before*.” *(Mary, Part A)*

The other 2 other subjects who initially used EMG-PR for grip select with Prototype 2 (1 TH, 1 TR) also experienced technical problems and changed to pressure transducer during training. They both expressed satisfaction with this manual mode of control. These types of problems led prosthetists to discontinue utilizing EMG PR for control of grip select. Thus, the 5 final subjects in the study used pressure transducers for grip selection without attempting to control it through EMG PR.

### Contextual factors

The study analysts identified a variety of personal and environmental factors that may have influenced subjects’ experience and reporting of their experience. Personal factors, including low frustration tolerance, interfered with learning and willingness to engage in training visits for several subjects. One subject clearly had difficulty in comprehending interview questions, as evidenced by several responses that did not address the questions asked. Several subjects, as mentioned above, had prior experience with the foot controlled DEKA Arm or with EMG-PR control of their personal device. These subjects may have had certain advantages over others in that they had less new information to learn. Residual limb pain, skin problems and intermittent swelling interfered with prosthesis wear time in several cases. It is possible that muscle atrophy or limited residual limb musculature due to extremely short residua or amputation approach may have made it more difficult for some subjects to utilize EMG-PR signals as compared to others.

Environmental factors, such as the subjects’ level of rapport with staff; personal issues creating a chaotic home environment, and health concerns for other family members, impacted subjects’ willingness and ability to engage with study activities. A major hurricane with flooding created a long disruption in training for one subject; and very hot and humid summer weather impacted another subject during Part B, who could not tolerate wearing any prosthesis due to excessive sweating.

## Discussion

Our work adds to the understanding of user experience of EMG-PR control of upper limb prostheses by providing an in-depth analysis of participant perspectives on using a commercially available EMG-PR control system that had been adapted to interface with the multi-DOF DEKA Arm. This system enabled EMG-PR control of up to 4 functions of the RC DEKA Arm, and up to 6 functions for users of the HC DEKA Arm. These functions included powered degrees of freedom of the prosthesis and toggle grip selection to enable switching of the DEKA Arm’s pre-programmed grip pattern. Our study also compared and contrasted participants’ views on EMG-PR control and the DEKA Arm as compared to the controls used with their personal prostheses, the attributes of their personal prostheses, and IMU foot controls of the DEKA Arm.

Although EMG-PR control of upper limb prostheses has been studied for decades, few clinical investigations have compared this control method to other control methods. One in-laboratory study, and one take home study, [20] compared clinical outcomes, such as dexterity and activity performance, for transradial amputees who used two or three DOF prostheses. These studies involved very small samples and reported mixed results regarding superiority of one control type over the other. A third study, which used a randomized control design, compared EMG-PR control to direct myoelectric control in TH amputees with TMR who had participated in a 6-8 week home trial [20]. In that study, Hargrove et al reported that functional outcome metrics improved after home use. Furthermore, and of most relevance to our study, they found that 7 of 8 subjects preferred EMG-PR to direct myoelectric control of the same prosthesis. However, they did not provide any detailed qualitative data or explore the reasons why users indicated that they preferred this control method.

However, findings from our study are not directly comparable to prior studies of EMG-PR control of upper limb prostheses because of the greater number of DOF of the DEKA Arm. In contrast to Hargrove et al, we found that the majority of prosthesis users who completed an in-home trial expressed a preference for the controls of their personal prosthesis, rather than the EMG-PR control. While our study demonstrated that EMG-PR is a viable option for control of the DEKA Arm or other multi-DOF devices, it also illustrates challenges using this method with multi-DOF devices.

We found that only 29% of home users of the EMG-PR controlled DEKA Arm stated that they wished to receive one in the future, and another 29% thought that they might want to receive one. Participants in our study made repeated remarks about the complexity of the control system and the challenges of calibrating due to numerous DOF, the added challenges of using a prosthesis that many perceived was heavy, and the need to repeated calibrations. Although some participants recognized the functional advantages of using the system, others did not. Most commented that they became fatigued when using this controls scheme, and reported that fatigue negatively impacted their ability to use the EMG-PR proficiently. Participants also described a process of learning and acclimation and recognized the need for sufficient prosthetic training. Similar to any surface myoelectric control, it appeared that EMG-PR control was susceptible to degradation caused by excessive sweating, and changes to prosthesis fit.

Others have raised concerns about the clinical robustness of surface EMG-PR due to problems with electrode shifting, limb orientation, and degradation of signals with repeated activation.[21] These concerns have led to many attempts at improving robustness of classifiers, as well as recommendations for training EMG-PR by maximizing sources of variation during the calibration (i.e. controls training) process, using visual feedback, and focusing on phantom limb movements [16, 22–24]. While most of our team was new to training participants using this control methodology, our team, with guidance from Coapt LLC specialists, attempted to utilize this training advice in our protocol to improve robustness of the EMG-PR controls. Nevertheless, our data shows that many of the concerns about reliability and robustness of EMG-PR remain. Others have recognized that training of EMG-PR (calibration) aimed to maximize variation is potentially burdensome and “prohibitively intensive.” [21] Feedback from our subjects demonstrates that many agree that calibrating in many positions is burdensome.

The 3 participants in our study who had experienced both control types had mixed viewpoints on EMG-PR compared to IMU controls of the DEKA Arm. Our findings did not indicate, as we had initially expected, that operating the DEKA Arm using EMG-PR would be easier to learn and less cognitively demanding than operating the device using IMU controls. Additional analyses of quantitative metrics directly comparing participants who used EMG-PR to those who used IMU controls are currently underway and will be published elsewhere.

Our study has several limitations. The study was not designed to assess the effectiveness of the training approach for use of the DEKA Arm and EMG-PR control. We cannot say with certainty whether or not our study participants would have had more favorable views of EMG-PR control of the DEKA Arm if they had been given additional training and time to acclimate. Despite the fact that all participants rated the amount of training that they had received as “just right”, we found that 4/7 who used the system at home rated their overall skill level as poor or fair. This finding suggests that additional training may have been beneficial. We do not know how our training quantity compared to that provided in the Hargrove et al study, because that study did not describe the amount of training provided [20]. We recommend further study to identify the optimal timing and dosage of prosthetic training required to optimize results for complex multi-DOF devices like the DEKA Arm. Both of our participants with TH amputation emphasized the need for sufficient training to master the extra DOFs using EMG-PR control. The participant who felt he mastered controls completely received 30 hours of training. However, he was an experienced using EMG-PR, albeit with a prostheses that had fewer DOF. This finding suggests that more than 30 hours of training may be needed for similar individuals.

Like the participants in Hargrove et al’s recent study, the two TH amputees with a history of TMR in our study were very positive about EMG-PR as a control system. In contrast, person with TR amputation, who needed to control fewer DOFs had more mixed responses to EMG-PR. However, due to our small sample size and the fact that there were no TH participants who had not undergone TMR, we cannot conclude that TH amputation level in and of itself is associated with greater satisfaction with EMG-PR compared to TR amputation level. Because of small sample sizes within sub-groups, we did not try to contrast the experiences of participants by years of prosthesis use, or type of prosthesis used at baseline. Future studies could examine differences in user experience within a variety of sub-groups.

Surface EMG-PR is thought to be more robust than direct EMG control because information from patterns of muscular recruitment derived from multiple electrode sites is used. Our findings suggest that prosthetic socket fit and changes in electrode contact within the prosthetic socket may have influenced the consistency of EMG-PR controls. That, said, we did not utilize any standardized metrics to evaluate prosthetic fit in our study, and cannot quanitfy the extent to which problems in control consistency stemmed from socket fit, not the control system in and of itself. Given that fitting of upper limb prosthetic sockets is more of an art than a science and that limb volume and shape may fluctuate which weight gain, fluid retention, and hot and humid conditions, there may be inherent vulnerabilities in utilizing surface EMG-PR systems. We believe that the quality of the prosthetic services delivered in our study were well above what would be available in the wider clinical community. Our study included several very well trained upper limb prosthetists who had expertise in fitting persons with upper limb amputation. Future studies could evaluate the relationship between prosthetic socket fit, consistency of electrode contact, and EMG-PR control consistency.

We found it challenging to disentangle participant preferences for control scheme from the characteristics of the DEKA Arm itself. There are unique aspects of the DEKA Arm compared to other prostheses These factors (such as number grips, wrist design, number of DOFs, heavier weight, required external wires and battery pack) may have impacted users (both negatively and positively) and thus colored their perceptions of overall prosthesis desirability and functionality. Given that ours is the first study to carefully describe user experience with EMG-PR controls, we were not able to identify or measure all relevant confounding/contributing issues. Future studies of EMG-PR might include quantitative metrics on the effect of the system on battery life.

Our findings are specific to EMG-PR control of the DEKA Arm and not EMG-PR control more broadly. We cannot say whether or not our participants would have preferred EMG-PR for control of their personal devices, or other devices with fewer DOF. We also cannot be sure if the fatigue associated with using EMG-PR might have been less of a factor with a lighter weight device. In our view, some amount of physical fatigue would be expected in the early phases of training as users cope with increased muscular demands.

We believe that our participants’ experience was impacted by technical issues originating in the EMG-PR/DEKA interface. Our study was the very first to test this new interface, and thus we identified compatibility issues, which no doubt would be addressed in future prototypes. EMG-PR control of grip select was considered by Coapt LLC to be an experimental feature. To our knowledge, no other research teams have attempted to toggle grip select with EMG-PR. This was inherently difficult, given that there is no natural physiological movement that is linked to this action. This may explain, in part, why there were so many issues using EMG-PR for this function.

## Conclusion

This study provided an in-depth description of the user experience of operating the multi-DOF DEKA Arm using EMG-PR control. Major aspects influencing the user experience related to the complexity of using EMG-PR system, the process of calibrating, and the functional benefits of using EMG-PR with the DEKA Arm. These aspects were largely explained by the technical issues, muscle fatigue, prosthesis design, technical issues with our EMG-PR prototype, training and acclimation. Factors influencing the user experience included training and acclimation, fatigue, aspects of the prosthesis design such as the weight, technical issues requiring repairs, changes to the controls. Broader contextual factors, both personal and environmental also impacted the user experience. An understanding of the patient experience of using EMG-PR to control a DEKA Arm, and factors that may affect that experience will be helpful for clinicians and patients who are choosing prosthetic control and hardware options.

We conclude that operating the multiple DOFs of the DEKA Arm using EMG-PR is more difficult to learn and no less cognitively demanding than operating the device using IMU foot controls. We found that the majority of participants expressed a preference for the controls of their personal prosthesis rather than the iteration of EMG-PR controlled DEKA Arm used in this study. Most home users were also positive about the future potential of EMG-PR control, or about the control scheme used in our study given further development. Both participants with TH amputation and a history of TMR, were particularly enthusiastic about EMG-PR for the number of DOFs required to operate an HC DEKA.

Our conclusions are limited to the prosthesis/control combination that we tested. Future studies are needed to evaluate whether the iteration of EMG-PR we tested would be experienced differently by users if it were interfaced with a prosthesis other than the DEKA Arm, particularly one with fewer DOFs.

## Supporting information

S1 File Appendix A: Semi-structured interviews. (DOCX)
