# Supplementary material for: User experience of controlling the DEKA Arm with EMG pattern recognition
Source: PLoS One. 2018 Sep 21;13(9):e0203987. doi: 10.1371/journal.pone.0203987 (PMC6150511; doi:10.1371/journal.pone.0203987)
Supplement: S1 File — (DOCX) [file pone.0203987.s001.docx]

**Appendix A: Semi-structured Interviews**

**Part A**

1. Please comment on your ability to put the Arm on, and take it off by yourself.
   1. If you could not put the Arm on or take it off by yourself, what would you need to be able to do so by yourself?
2. How confident are you in your ability to hold onto an object using the DEKA hand while walking? Do you prefer to put the Arm in standby when walking while holding an object?
3. Please comment on the look of the entire DEKA Arm system.
4. As compared to your sound side, how did you feel about the proportion (length, position of the joints) of the DEKA Arm?
5. Please comment on the look of the DEKA hand – separate from the whole Arm system.
6. Are you comfortable wearing the DEKA Arm in public right now?
7. If you have another prosthesis, would you prefer to wear it in public instead of the DEKA Arm or would you prefer to wear the DEKA Arm? Why or why not?
8. Please comment on the sounds that the DEKA Arm makes.
   1. Would you be willing to have these sounds heard in public? Why or why not?
9. Please comment on the overall reliability and durability of the DEKA Arm system.
10. Please comment on the need for service of the whole Arm system (if applicable) experienced in the study
11. How do you think that the experience of being in this study impacted you as a prosthetic user?
    1. Are you using your own prosthesis more/less/or the same as before you began the study?
    2. If you are not a prosthesis user, how do you feel about using a prosthesis now?
12. What did you like most about the DEKA Arm?
13. What did you like least about the DEKA Arm?
14. As of right now how long can you comfortably wear the DEKA Arm without having to doff the prosthesis?
15. FOR CURRENT USERS ASK: How does this compare to the amount of time you can comfortably wear your current prosthesis?
16. Please comment on faults? Was the frequency of faults acceptable to you? How easy or difficult were they to correct?
17. Please comment on the battery life of the:
    - 1. External battery;
      2. FOR HC USER**:** Internal battery;
18. IF A CURRENT PROSTHESIS USER ASK: Compared to your existing prosthesis, please comment on the DEKA Arm system in terms of:
    - - 1. controls
        2. weight
        3. cosmesis/appearance
        4. advantages

disadvantages

1. For subjects who did not complete the Mid study survey, please ask:
2. Do you feel that practice with the Virtual Reality Environment (VRE) made learning to control the Arm easier? (Yes/No) Please explain.
3. What type of training activities so far have been most useful to you?
4. What type of training activities have been least useful?

***The next questions are about the use of the EMG pattern recognition controls (Coapt).***

1. Please comment on using the Coapt system to control the DEKA Arm prosthesis.
2. How well do you feel you have acclimated to this system? (“not at all,” “somewhat,” “mostly,” or “completely”)
3. IF A CURRENT PROSTHESIS USER:
   1. How does it compare to your current control system?
   2. Do you feel this system makes it easier to perform daily activities than your current control system?
   3. Do you feel it is more intuitive than your current system? Please explain your answer.
4. If the subject participated in the optimization or home study and used IMUs ask***:*** Please compare Coapt control to IMU control of the DEKA Arm. Which method do you prefer and why?
5. If you currently use a Coapt system (or pattern recognition control) for your own prosthesis how did using and calibrating the Coapt system with the DEKA Arm compare to calibrating and using your own prosthesis?
6. How did the weight of the DEKA Arm affect your ability to control the prosthesis using pattern recognition controls? Please explain.
7. Do you feel confident that you will be able to calibrate the Coapt system at home?
   1. Are you able to calibrate the Arm by looking only at the wrist display and/or Coapt LED lights to monitor grip select and mode select?
   2. Do you feel watching a video or screen display was helpful while calibrating, and would you prefer having access to a video to use at home?
8. Muscle fatigue sometimes causes issues with pattern recognition control.
   1. Please comment on whether you experienced this during training, and if so, talk about the strategies you used to address it?
   2. If you are taking the DEKA Arm home, what strategies will you use if your muscles become fatigued while performing an activity?
9. Were you able to control grip select through the Coapt system or did you use a pressure switch to control grip select?
   1. If you used Coapt for grip selection, please comment on the ease of using grip select.
   2. If you used Coapt to control grip select, how easy is it for you to toggle grips in both directions / in one direction (if applicable)?
   3. How useful are the Coapt LED lights when toggling grips? Please explain.
10. FOR HC USERS: Please comment on the ease of using mode select with the Coapt system. How did you control mode selection?
11. Were you able to use the Coapt LED lights to distinguish Arm Mode from Hand mode?
12. If you are taking the Arm home, do you feel confident troubleshooting any issues with Coapt should they happen at home?
13. Is there anything else you’d like to add about being in this study, using the Coapt system, or the DEKA Arm that we haven’t covered toda7

**Part B**

1. Thinking back to the three months of using the DEKA Arm at home, did you use the

Arm more than you expected, less than you expected or about as much as you

expected? What factors made you wear the Arm more or less than you expected?

2. Did the amount of time that you wore the DEKA Arm change over the past three months? If so, how and why?

3. Please talk about anything that has surprised you either positively or negatively about using the DEKA Arm at home.

4. Based on your experience using the DEKA Arm at home, what advice do you have for new home users of the DEKA Arm?

5. Please comment on the weight of the DEKA Arm system.

6. Please comment on ease of donning or doffing the DEKA Arm system.

7. Please comment on the socket fit.

8. As compared to your sound side, how did you feel about the proportion (length, position of the joints) of the DEKA Arm?

9. Please comment on the look of the entire DEKA Arm system.

10. Please comment on the look of the DEKA hand separate from the whole Arm

system.

11. Are you comfortable wearing the DEKA Arm in public right now?

12. ASK IF SUBJECT IS A PROSTHESIS USER: If you have another prosthesis, do

you prefer to wear it in public instead of the DEKA Arm or do you prefer to wear the

DEKA Arm? Why or why not?

13. Please comment on the sounds that the DEKA Arm makes.
 a. How comfortable are you with having these sound heard in public?

14. Please comment on the overall reliability and durability of the DEKA Arm system.

15. Please comment on the need for service of the Arm system that you experienced during the study.

16. How do you feel about the study ending? What will you miss most about not having the DEKA Arm at home?

17. How do you think that the experience of being in this study impacted you as a prosthetic user?

a. Are you using your current prosthesis more/less/ or the same as before you

began the study?

18. What did you like most about the DEKA Arm?

19. What did you like least about the DEKA Arm?

20. As of right now how long can you comfortably wear the DEKA Arm without having

to doff the prosthesis?

IF A CURRENT PROSTHESIS USER ASK:

How does this compare to the amount of time you can comfortably wear your current prosthesis?

21. Please comment on the IMUs as a means to control the DEKA Arm prosthesis.

a. How well do you feel you have acclimated to using foot controls? (“not at all,”

“somewhat,” “mostly,” or “completely”)?

22. Please comment on the walk detect feature of the DEKA Arm. How did walk detect

work during extended walking periods or when you were walking fast?

a. Did you use Standby when walking with the DEKA Arm, instead of walk

detect? If so, please explain why and in what circumstances?

23. Please comment on faults? Was the frequency of faults acceptable to you? How

easy or difficult were they to correct?

24. Please comment on the battery life of the

a) external battery?

b) FOR HC USERS: internal battery;

25. IF a CURRENT PROSTHESIS USER ASK: Compared to your existing prosthesis,

please comment on the DEKA Arm system in terms of:

a) how it is controlled

b) weight

c) cosmesis/appearance

d) advantages

e) disadvantages

***The next questions are about the use of the EMG pattern recognition controls (Coapt).***

26. Please comment on using the Coapt system to control the DEKA Arm prosthesis.

1. How does it compare to your current control system?
2. Do you feel this system makes it easier to perform daily activities than your current control system?
3. Do you feel it is more intuitive than your current system? **(IF A CURRENT USER)**
4. How well do you feel you have acclimated to this system? (“not at all,” “somewhat,” “mostly,” or “completely”)

27.How did the weight of the DEKA Arm affect your ability to control the prosthesis using pattern recognition controls? Please explain.

28. Please comment on calibrating the Coapt system:

1. Were you able to calibrate the Arm by looking only at the wrist display and / or Coapt LED lights?
2. While at home, did you ever decide not to use of the DEKA Arm because you were having problems with PR controls? If so, how often did this happen?
3. About how often did you recalibrate the system while at home?
4. Did you need to recalibrate after each donning of the socket?
   1. Please comment on anything you learned about what affected the success of making satisfactory calibrations.

29. If you currently use a Coapt system (or pattern recognition for your own prosthesis how did using and calibrating the Coapt system with the DEKA Arm compare to calibrating and using your own prosthesis?

30. Regarding reliability and troubleshooting the Coapt system:

1. Was the frequency of need for repair/adjustments by the prosthetist to the Coapt system acceptable to you? Please explain why or why not.
2. Please comment on your experiences troubleshooting the Coapt system at home without the assistance of a prosthetist.
3. Over time, did the frequency of need for repair or adjustments to the Coapt system affect your willingness to wear and use the Arm at home? If so, please explain.
4. If there were long interruptions in use of the Arm due to problems with the Coapt system, how did this affect resuming use?
5. How did the frequency of need for repair/adjustments to the Coapt system affect your interest in having a DEKA Arm integrated with the Coapt system in the future, if it became available?

31. Given your experience, would you recommend use of the

Coapt system for control of the DEKA Arm?

a.Why or why not?

1. Is there anything else you’d like to add about being in this study, using the Coapt system, or the DEKA Arm that we haven’t covered today?
